# Wearable Solutions for Patients with Parkinson’s Disease and Neurocognitive Disorder: A Systematic Review

**DOI:** 10.3390/s20092713

**Published:** 2020-05-09

**Authors:** Asma Channa, Nirvana Popescu, Vlad Ciobanu

**Affiliations:** Computer Science Department, University POLITEHNICA of Bucharest, RO-060042 Bucharest, Romania; nirvana.popescu@upb.ro (N.P.); vlad.ciobanu@upb.ro (V.C.)

**Keywords:** wearable sensors, Parkinson’s patients, Parkinson’s disorder, neurocognitive disorder, rehabilitation exercises

## Abstract

Prevalence of neurocognitive diseases in adult patients demands the use of wearable devices to transform the future of mental health. Recent development in wearable technology proclaimed its use in diagnosis, rehabilitation, assessment, and monitoring. This systematic review presents the state of the art of wearables used by Parkinson’s disease (PD) patients or the patients who are going through a neurocognitive disorder. This article is based on PRISMA guidelines, and the literature is searched between January 2009 to January 2020 analyzing four databases: PubMed, IEEE Xplorer, Elsevier, and ISI Web of Science. For further validity of articles, a new PEDro-inspired technique is implemented. In PEDro, five statistical indicators were set to classify relevant articles and later the citations were also considered to make strong assessment of relevant articles. This led to 46 articles that met inclusion criteria. Based on them, this systematic review examines different types of wearable devices, essential in improving early diagnose and monitoring, emphasizing their role in improving the quality of life, differentiating the various fitness and gait wearable-based exercises and their impact on the regression of disease and on the motor diagnosis tests and finally addressing the available wearable insoles and their role in rehabilitation. The research findings proved that sensor based wearable devices, and specially instrumented insoles, help not only in monitoring and diagnosis but also in tracking numerous exercises and their positive impact towards the improvement of quality of life among different Parkinson and neurocognitive patients.

## 1. Introduction

Nowadays, millions of people are bearing cognitive deterioration [1]. There is an extensive range of neurocognitive disorders specified as Alzheimer’s disease, Parkinson’s disease, Traumatic brain injury, Lewy body disease, Vascular disease, Frontotemporal lobar degeneration, etc. [2,3]. Comparatively, Parkinson’s disease (PD) is the second most frequently observed neurodegenerative disease [4]. Around seven to ten million people in the world have Parkinson’s disease. The examination of Parkinson’s disease is a tricky one; research has been done for decades and still there is no robust test that serves as the best since Parkinson’s disease signs and symptoms vary from person to person and its features are very similar to other illnesses [5]. PD can be investigated incorrectly and can be interpreted with other diseases. Characterization of tremors in hands and gait features plays a vital role in diagnosis and long-term monitoring of Parkinson patients.

Typical gait disorders of PD can be illustrated as freezing of gait (FOG), stooped posture, shuffling steps, festination, and falling [6]. Current clinical solutions for detecting gait use motion sensing technology i.e., camera-based optical motion-capture system, markers attached with body, force plated on the ground to measure pressure, equipped treadmills with different sensors and for quantifying tremors, laser displacement sensors, and electromyography (EMG) systems [7,8] are used. For diagnosis, assessment, and monitoring, there is a need for continuous data from patients and the solution lies in wearable technology [9]. The role of wearables is multifold, and it starts from continuously capturing the motion data [10] that helps in diagnosis, monitoring, and tracking the changes in compliance with exercise training, boosting, and also from clinical trial data. It does not only monitor PD symptoms but helps in improving them. Hence, the aim of this systematic review is to find the use of sensor based wearable devices in the diagnosing and monitoring of PD patients, analyzing their role in enhancing PD patients’ quality of life (QoL) and clearly identifying how wearables can help in finding the results of various fitness and rehabilitation exercises.

## 2. Method

### 2.1. Data Collection

**Figure 1:**
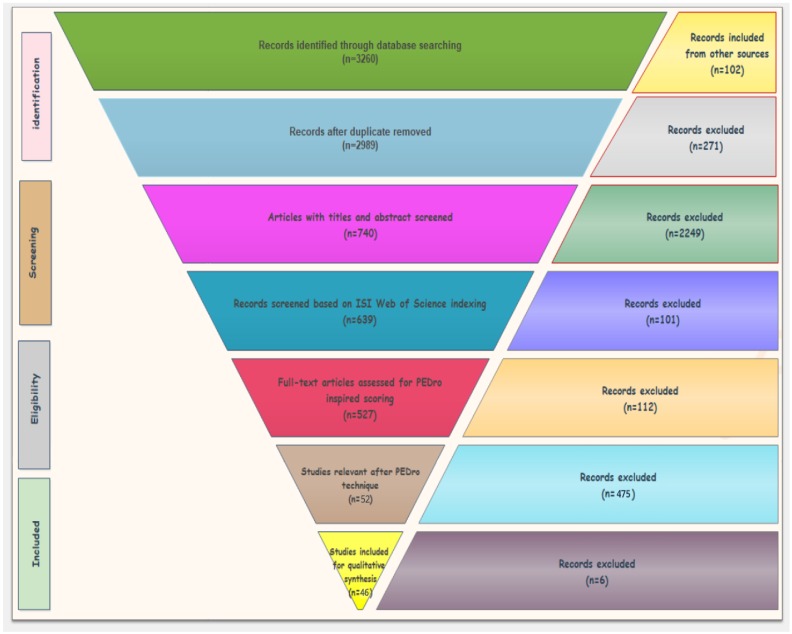
PRISMA adapted flow diagram used for the articles’ systematic selection.

**Figure 2:**
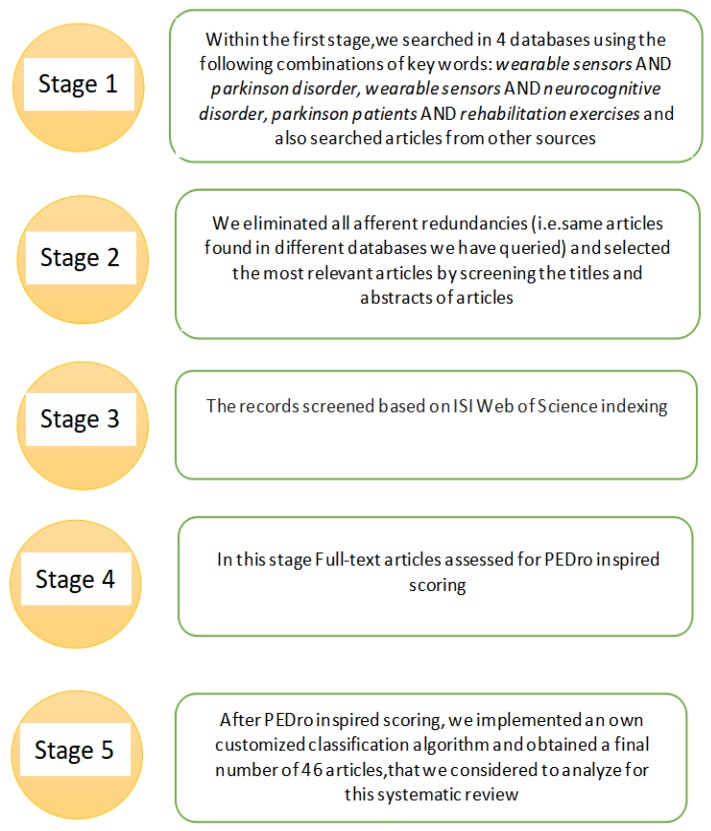
Stages adopted for the systematic selection of articles.

Our systematic literature review collected publications from January 2009 to January 2020 from the following databases: Elsevier, IEEE Xplorer, and PubMed/Medline. Our search for finding relevant articles is comprised of five stages based on Preferred Reporting Items for Systematic Reviews and Meta Analyses (PRISMA) as shown in Figure 1 and Figure 2. In the initial stage, we used three combinations of keywords: “wearable sensors AND Parkinson disorder”, “wearable sensors AND neurocognitive disorder”, “Parkinson patients AND rehabilitation exercises” in the three databases mentioned above, and we got around 3158 articles as result. We also added 102 relevant articles found from other sources with these keywords and hence we got 3260 articles as shown in Table 1.

In the second stage, we initially removed 271 duplicates since the same articles were found in different databases, and we also removed articles on the basis of careful analysis of titles and abstracts. In this way, 740 records were obtained as shown in Table 2. In the third stage, we filtered out the articles and considered the ones which are ISI indexed. Based on this criteria, we got 639 articles as shown in Table 3.

In the fourth stage, we have developed our own customized PEDro inspired scoring that classified the most relevant articles fitted for our system review. In PEDro, five statistical indicators are set as answers to the following questions:

1. Does this article prove that wearable sensors can be used to quantify recovery automatically in a laboratory setting?
2. In this article, were wearable sensors used to monitor people remotely in their home?
3. How many patients/subjects participated in this study?
4. Does this article discuss the exercise rehabilitation that can enhance the recovery and fitness in (PD) patients?
5. Is this a Survey/Review article?

In this way, the articles were analyzed by means of the answers to these above presented questions and we selected those publications that obtained at least four points (“fair”/“high” quality), by applying the following grading criteria as represented in Equations (1) and (2):

(1) $$Qn^{*}=\sum_{r=1}^{n=5}Qr$$

(2) $$Qn^{*}=\left\{\begin{array}{cc} Qn^{*} & Qn^{*}<4\\ 4 & Qn^{*}\geq 4 \end{array}\right.$$

*Q3* refers to the number of human subjects included in the study for each article, and it is shown in Table 4. However, this standard does not apply for review articles—for survey papers, a different perspective is set, following the same calculations. In this case, the references’ quality was considered as depicted in Table 5. For each criterion, the maximum number of points possible to be obtained was 1.

A balance between the newly published articles with less number of citations and the older ones which have more citations have been considered further. For this reason, a new tailored formula is developed. Its purpose is counting the number of citations obtained each year for each article starting from the year in which the article was published. Some steps should be mentioned at this point. In the first stage, Equation (3) gives the number of citations registered every year for one candidate article:

(3) $$PYCi=\frac{TCi}{2020-Yi}$$

In the above equation, PYC represents the number of citations per year, TC shows the total number of citations, and Y specifies the year when the article was published. The equation takes into account the idea that some articles are published in different years and the number of citations depends on this aspect. Thus, the newer articles have a different weight than the older ones. In this context, Equation (4) presents the method of computing the absolute value of quality Qi* for an article from the citation point of view:

(4) $$Qi^{*}=\frac{5*PYCi}{max_{j=1\ldots n}\left(PYCj\right)}+\frac{6-min(2020-Yi,6)}{2}$$

As it will be seen in Equation (5), all of the absolute values should be inside the interval [0:5]. This final value *Qi* has been chosen to range the maximal score up to 10 because the total score of each article is, in fact, the average of each statistical indicator multiplied by 2:

(5) $$Qi=\{\begin{array}{cc} Qi^{*}, & Qi^{*}<5\\ 5, & Qi^{*}\geq 5 \end{array}$$

All these selection criteria have been applied according to the discussed equations and the final number of articles remained to be analyzed became 46. The systematic review will focus further on the analysis of this reduced set.

### 2.2. Method of Analysis

After we created our collection of the most relevant articles by the quantitative analysis, the next phase regards the qualitative analysis of the remaining 46 articles. Considering the main purpose of this systematic review, we further investigated them following some relevant research questions as:

1. Does motor disability play any role in altering the quality of life of PD patients?
2. Do wearable insoles help in diagnosis, monitoring, and rehabilitation of PD patients?
3. Which gait or fitness exercise impact the quality of life?
4. Which fitness tests lead in proper diagnosis of balance, walking, and aerobics fitness of PD patients?

## 3. Outcomes of the Systematic Review

This section is divided into four parts. Each of them revolves around the main aims of this systematic review.

### 3.1. Early Diagnosis Wearables and the Effects of Motor Disorders on Quality of Life (QoL)

**Figure 3:**
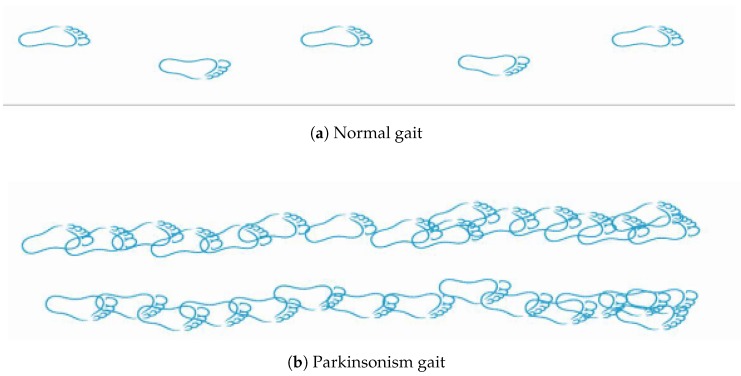
(**a**) shows Normal person gait and (**b**) shows PD patient gait.

Preponderance of Parkinson’s and overall motor symptoms affects the QoL of PD patients very badly [11]. Gait disorders are classified according to an accepted scheme and their associations to falls. Neuro-psychological measures and QoL have been explored for decades, a fact that proved that gait impairments significantly diminish QoL. The main motor disabilities faced by PD patients are elaborated in Table 6 and Figure 3. Gait disorders are the most common among PD patients, reducing the mobility in the daily life activities and becoming worse as disease advances [12]. The difference between normal and Parkinsonism gait can be seen in Figure 3. Hence, PD leads to major walking problems, causing falls and hence leading to long-term disability and independence loss.

The role of wearables in improving early diagnosis and monitoring is further analyzed considering the great improvements brought by the wearable devices proposed by so many researchers in this field. The most promising role can be played by the wearables which are less expensive, consume less power, are unobtrusive, and provide more accurate data in diagnosing, monitoring, and managing a rehabilitation process. Wearables in PD applications may be helpful in early diagnosis, tremor, body motor fluctuations, and home and long-term assessment as discussed in [13]. Currently, PD diagnosis relies on monitoring the motor and non-motor significance and usually doctors check the severity of PD patients disease by asking them to perform specific tasks and assign them scores based on Unified Parkinson Disease Rating Scale (UPDRS) or Movement Disorder Society-sponsored revision of UPDRS and Hoehn and Yahr scale. Many times, the result provides a 40% wrong diagnosis because of the inter-rater variability among different examiners. Early, prompt, and accurate diagnosis of PD may improve QoL and, regarding this, wearables play a fundamental role in helping clinicians perform early diagnosis and objective quantification. In [14], the researchers proposed a pedestrian dead reckoning (PDR)-based method using a smartphone. The accelerometer sensor in a smartphone monitors the changes of walking patterns of subject like PD and captures the gait characteristics, such as step length and step frequency. This early warning diagnosing tool gives about 98% accuracy of step length estimation. In [15], researchers introduced another early diagnosing wearable wireless system with a proprietary algorithm. The system is composed of IMUs attached on the patient’s lower limb. The system is tested on 20 subjects and showed promising results, the maximum error in stride number estimation was as low as three units.

Tremor is the most frequently observed symptom among PD patients. It appears in 70% of them. In [16], a custom-developed device (SNUMAP) is designed using an accelerometer and gyroscope, fabricated on a wrist module. The system is validated on 92 PD patients and showed more precise monitoring of PD tremors. In [17], a smartwatch is developed for PD tremor analysis which turns out to be very reliable, well-correlated with clinical scores, and well-accepted by patients for clinical follow-up. Bradykinesia is another symptom that severely affects QoL. In [18], the wearable IMUs are implemented to quantify whole body movements, producing Bradykinesia indices for walking (WBI) and standing up from a chair (sit-to-stand; SBI) and compute an objective score for whole body Bradykinesia.

The concept of monitoring patients in their own homes is the future trend in long-term monitoring. In this context, [19] proposed a multi-sensor monitoring unit (WMSMU) called PERFORM for monitoring, assessment, and management of patients. In [20], another system using IMUs and smartphone-based application served as an adequate gait training application in home infrastructure by improving postural balance and gait activity. The system gave auditory cueing to prevent or overcome FOG episodes. Similarly, the articles [21,22] proposed on-shoes wearable sensors and monitoring insoles that helps in gait assessment and monitoring. Thus, an optimal solution for improving monitoring and assisting PD patients lies in wearable technology which has been proven to be more flexible to be adopted in both clinical framework and in a home environment.

### 3.2. E-Health Wearables for PD Patients

The current demand of technology for PD appraisal, intervention, and rehabilitation varied in its requirements and can be counted from cost, usability, working, efficiency, design, and continuous quantitative and qualitative information [12]. In the beginning, clinical based scales were set to check motor symptoms severity which resulted in an uneven ratings and wrong measurements. The introduction of a smart environment such as body attached sensors, ubiquitous networking, and embedded sensors facilitates healthcare allied assistants to automatically monitor PD patients in real world environments. For instance, the researchers in [23] monitored full body tremor, which is one of the dominant symptoms among PD patients, using an inertial measurement unit (IMU) based motion capture system and detecting tremor against non-tremor dominant individuals among a group of 40 PD as well as from 20 healthy controls. In [24], the authors focused on another PD debilitating symptom that is freezing and discussed the variety of lightweight and wearable inertial sensors that may help in monitoring FOG (freezing of gate) in PD patients which uses dopaminergic medication. Similarly, in [25], the authors proposed a method for finding gait freezing events amid normal walking using skin conductance (SC) features and multivariate Gaussians.

The research study in [26] brings the key challenges in using wearable sensors i.e., data management, scalability, interoperability, standardization, security, and privacy and also proposed a smart glove in which flex sensors are attached to detect motor symptoms such as tremor, rigidity, and slowness of movement. The different endowed e-health wearables that assimilate contextual data are: DynaPort MiniMod Hybrid (worn on the lower back), Parkinson’s Kinetigraph (a wrist worn logger), a KinetiSense motion system (for dyskinesia measurements), ActivPAL, Stepwatch 3 (step activity monitor), Shimmer (records walking and turning), Mobi8Senior mobility monitor (SMM, Philips), SENSE-PARK system (for gait, hypokinesia, dyskinesia, sleeping), GAITrite (gait analysis systems), Opal (to asses quality of turning), Actigraphs (to monitor sleep), and also cueing devices such as auditory cueing devices, visual cueing devices, and somatosensory stimuli devices [27,28,29,30,31,32,33]. All of these devices help in feature classifying of PDs determining the disease severity, motor impairment, and also the improvements after the exercises.

#### 3.2.1. Wearable Device(s)

Most of the wearable devices are developed based on inertial sensors that are comprised of an accelerometer and gyroscope. Accelerometers are used to measure accelerations but are unable to measure the rotations or angles. Therefore, these can not help with detecting the turns during walking activities. On the other side, gyroscopes serve in detection of angular velocity of body and also there are less chances of mechanical noise than in an accelerometer’s case, hence turning is better assessed during motion. Being critical with gyroscopes, it can be mentioned that their drawback relates to the high power consumption during long-term recording. Battery life, type, and number of inertial sensors, sampling rate, recording and processing time, and, most importantly, the learning algorithm is the key factor that makes the difference between the accuracy and precision of wearable devices. In this section, we highlight the ones that assess motor symptoms of PD patients and offer insights in diagnosis, cueing, and testing.

In [27], the authors proposed a device named Opal, with a weight of 22 g. It is built using inertial sensors, a battery, and includes 8 GB of memory storage. The device is tested in a research study in which users wore three Opals, one on the belt and the remaining two inside shoes. Data were recorded at 128 Hz and later uploaded to a laptop. The data from this device help in assessing the quality of turning. The researchers in [29] introduced a Kinesia system that consists of a software application, a hybrid sensor worn on the finger, and an automated web-based symptoms assessment system. The patients wore the sensors on the index finger of the most affected hand. The assessment is based on five motor tasks each of 15 s to predict finger tapping, dyskinesia, hand opening and closing, and also the postural tremor. The Parkinson’s Kinetigraph from [30,32] is used to measure the wrist movements and is worn like a wrist bracelet. It weighs around 35 g, and it has a three-axis iMEMS accelerometer (ADXL345 analog device) that records acceleration with a value of ±4 g at sampling frequency of 50 samples/s. The device is developed using a digital microcontroller with flash memory along with a rechargeable battery. The sensor apprehends the Bradykinesia and dyskinesia values in a two-minute span for 10 days using a fuzzy logic algorithm. The device is preferred to be worn at the most affected limb of PD patients. The GAITRite in [30] is a walkway with a length of 4.6 m connected with Windows XP through the serial port. The thickness of passageway is 1/800 and has 16,128 sensors attached between two layers of vinyl and a rubber. It helps with demonstrating Bradykinesia and can be used in the replacement of conducting a traditional timed test such as a TUG test or filling questionnaires from PD patients [28].

Actigraphs are movement detectors, which are constructed with accelerometers and a memory for recording the movements for few weeks. The programs are developed to determine the levels of rest/movement, rhythmic parameters, and running/sitting parameters. In [27], Actigraphs, in the form of wrist worn activity sensor, are used for sleep monitoring. The KinetiSense motion system in [30] served for accurate measurements of tremors, Bradykinesia, and dyskinesia. It is built using accelerometers and gyroscopes that are attached on three areas of the body. This system is considered to be beneficial in developing new therapies. Stepwatch 3 is called an ankle acceleromater, fixed on the leg for counting stride rate. It is one of the devices with most valid and reliable results in monitoring ambulatory activity as discussed in [27,30]. ActivPAL™ in [31] is a small, lightweight activity monitor device that has a uni-axial accelerometer fixed on the upper thigh, at 10 Hz sampling frequency. The raw data in form of spreadsheet are exported for further analysis in MATLAB. The SENSE-PARK System in [31] has a set of inertial sensors (three are used during daytime and one in night phase) that helps in detecting movements of PD subjects i.e., FOG, dyskinesia, tremor, and sleep using an algorithm. The system also has a Wii balance board for collecting information such as body weight and sway.

In the study [31], SHIMMER sensors are introduced. These are kinematic sensors developed with gyroscope and an accelerometer that performs sampling at 102.4 Hz. These are attached in the form of elastic bands. The recorded data are transferred to computer wireless using Bluetooth. A total of 21 features are selected for reliability analysis from the recorded data. Another ambulatory assessing device is Mobi8 proposed in [31], which is a multichannel data logger with a dimension of 11.4 × 9.8 × 3.7 cm${}^{3}$, weighs up to 165 g, has a 3D sensor (Analog Devices ADXL330), and is worn on the lower back. It records anterior-posterior, vertical, and mediolateral, respectively. For finding daily life activities such as walking, the Senior Mobility Monitor (SMM) [31] was implemented. It is comprised of an accelerometer and barometer. The data are sampled at 50 Hz for the accelerometer and 25 Hz for the barometer. SMM is required to be worn at sternum height. The data are analyzed using a wavelet-based decision tree algorithm in MATLAB^®^, version 2013a. DynaPort MiniMod Hybrid in [32] weighs 74 g and has dimensions of 87 × 45 × 14 mm. It is attached on a belt on the back to show lower body movements in performing DLA. The device consists of accelerometer with a limitation of ±2 g, a resolution of ±1 mg, and a triaxial gyroscope. The readings are stored on an SD card at a frequency of 100 Hz and transferred in MATLAB Software for further analysis of gait features. The system aids in monitoring and classifying the quality and quantity of gait in PD faller and non-faller groups.

The study [33] provides a technological review on available wearable cueing devices, highlighting the current auditory, visual, and somatosensory cueing devices. The auditory cueing devices include Android applications based on Google Glass, GaitAssist (equipped with two inertial sensors and a smartphone with android application and wired headphones), FoG detection devices with wireless ear sets, Metronome Peterson bodyBeat and Metronome SDM300 SAMICK (Peterson Electro-Musical Products, Inc., Alsip, IL, USA), and devices with a movement sensor enabled with Bluetooth and wired headphones. These devices produce a typical and distinctive sounding tones (i.e., tap, tick, click, and beat) in beats/minute. In this way, it generates temporal information such as step interval, through the rhythmical beat. Some visual cueing devices are: Laser shoes, Smart Gait-Aid (Android app on binocular smart glasses), and Visual-auditory walker. These devices demonstrated that visual stimuli can diminish the FOG occurrence during walking. Parallel patterns aid in conveying spatial parameters’ information, such as step duration. Some somatography cueing devices are: CueStim (two channel electrical stimulator), Vibrating waistband, and a Vibrating system named VibroGait.

#### 3.2.2. Insoles Models and Technical Features

According to primary research studies, there are two ways to evaluate motion activities of PD patients: subjective and objective. The subjective methods are based on questionnaires, UPDRS criteria, or Hoehn and Yahr scales in which there are more chances of getting an incorrect evaluation and error in scaling. The objective assessment is based on a huge variety of body worn sensors such as accelerometers, gyroscopes, magnetometers, force sensors, etc. that detect each fine movement and angles of a person performing (daily life activities) DLA, but the criterion validity of these wearable e-health devices vary from one to another. Not all the aforementioned devices in [27,28,29,30,31,32,33] are appropriate for daily routines in people with Parkinson’s disorder, and it is hard to find a single wearable device for diagnosis, monitoring, and rehabilitation of PD. A systematic review in [34] provided a potential solution for continuous and unobstructed appraisal of Parkinson’s patients that resides in smart insoles.

There is no doubt that humans wear shoes continuously, and the insoles are much cheaper than other wearable devices commercially available in the market. Table 7 presents an overview of the insole models discussed in papers [35,36,37,38,39]. These insoles can extract gait features and help in classification of PD stages and in daily monitoring for rehabilitation purposes. The results of the comparative study [34] emphasize that the data validity of smart insoles is 75% to 100% accurate, 75% to 100% precise, and the specificity lies between 73% to 100%. In this way, we can say that smart wearables [40] allow quantitative, objective, and reliable evaluation of motor activities.

#### 3.2.3. Algorithms for Analysis of Gait Pattern

However, besides the acquisition of a robust data set from wearable insoles, another noteworthy aspect regards the algorithms that accurately learn from the data and also accurately predict [41]. Machine learning algorithms are currently considered as the pervasive part of the smart environment, but PD data are different in a more sophisticated way from the traditional clinical data as it is comprised of high-frequency continuous digital sensors readings of around tens of thousands per second.

ML algorithms such as random forest (RF), decision trees (DTs), logistic regression (LR), support vector machine (SVM), hidden Markov models (HMMs), naive Bayes, clustering algorithms, and neural networks (NNs) have been implemented successfully in medicine [32] and recently expanded to use them for sensor based PD motor assessment. A systematic study [42] revealed that ML algorithms depend on the data and on the features that are needed to be extracted as it is discussed in [43], where a TRIS (treatment response objective index) algorithm is discussed that examines clinical effectiveness with regard to dose response.

The outcome of studying these insoles models proved that smart insoles can identify either the subjects are sitting, standing, walking, or lying and can also differentiate between normal gait and Parkinsonism gait features. Last but not least, these can be useful to find the improvements in movement after exercises.

### 3.3. Clinical Effects of Rehabilitation Exercises on PD Patients

Research has proved that gait disorder creates disability and determines poor QoL because of the cardinal symptoms i.e., FOG (freezing of gate), tremors, and falls in people with PD. Systematic reviews and guidelines confirmed that the motor exercise is an effective method to rectify the gait and the overall physical functioning, improving QoL. Many fitness exercises like physiotherapy, center or home based workouts, treadmill training, visual or acoustic cueing and upper and lower body strength exercises have become an integral part of managing Parkinson’s disease. However, clinical trials have been unsuccessful in identifying which motion oriented gait exercise method can give the best results. In this systematic review, one of our objectives is to compare the results of different training activities [44,45,46,47,48,49,50,51,52] as elaborated in Table 8, and to assess the overall changes in movement related disorders and QoL.

“Exercise is medicine” [49] are the perfect words that fit for PD patients. Improvement in QoL appears to be the most general form of rehabilitation, even if it is resulted just based on home exercises, tailored center based training, robotic, or treadmill. Our study based on different reported research works reveals the fact that most of the training is conducted outside the home [53]. Home based exercise gives similar results to the instrumental based one, and it is applied to patients who are not in an advanced stage of PD. It is also observed that cognitive and psychological rehabilitation is just at the beginning, but it is an emerging area of research. The majority of interventions missed taking inter-professional approach planned without the consent of patients and conducted in a home environment. Considering all the interventions, it is observed that PD rehabilitation needs more concentration and research. Coordination between distinctive health care professionals and multidisciplinary support teams is needed to tackle the complexity of PD and more work is needed in this area [54]. Such multidisciplinary support must be tailored keeping the desires and objectives of each individual PD patient.

### 3.4. Fitness Test for Objective Evaluation of Rehabilitation

After selecting the type of exercise, the location, approach, and the intensity duration required to achieve motor benefits, the next relevant concern is how to find a robust evaluation test that correctly shows the results of rehabilitation. The assessment of motor activity in PD has been growing in recent years among research communities. Previous rehabilitation tests have varied with every individual, but researchers have considered the *timed up and go (TUG)* test as more efficient. In this test, individuals are asked to perform some tailored motor activities (i.e., sitting, in stood posture, turning, climbing) and the positive feedback proves the improvement of specific rehabilitation training. However, the TUG test gives promising results by just counting on the total time taken by patients to complete the task, and addresses some lower extremities’ dysfunctions. These two factors are not enough for evaluation of PD patients’ motor recovery. Hence, numerous tests have been proposed in recent years. Table 8 depicts the type of test for each research study used to predict the amount of benefits of each training.

In addition to TUG tests acquired using the wearable sensors, two other examination methods using wearable sensors are described in articles like [38,39]. In [38], the Ambulosono wearable works with an iOS GaitReminder App that produces auditory instructions while continuously recording step sizes via iOS gyro and accelerometers (after correcting the limb length, angular excursion, signal filtering, and drift). During the tests, the patients uniformly received a set of standardized auditory instructions, tailored by clinical guidelines, through wireless headphones, which contain verbal encouragement, specification of walking speed (e.g., comfortable or fast), and reminders of completed walking time. Step features collected via GaitReminder App show an average of <10% difference when it was tested against direct video measurements, and an analogous error rate was conjointly found once the App was used for treadmill walking or over-ground walking activities. A new generation of the Ambulosono sensor was designed, and it demonstrated a <5% error rate in gait measurement.

Another wearable based method is the combination of TUG and different subsystems. As TUG gives promising results in evaluation of lower extremity dysfunctions, the total time to finish the TUG does not give insight into turning and transitioning from standing to sitting actions. Hence, [39] highlighted the importance of segmenting the TUG into phases. In their study, they proposed that IMU data should be extracted from an iPad coupled with a custom built application. The Cleveland Clinic Mobility and Balance Application (CC-MB) could be used to segment the TUG into the subtasks. However, the study is in an infant stage and needs validation and more work to verify the results.

Table 8 clearly emphasizes the idea that currently the TUG test and the 6 or 10 min walking test are mostly considered for evaluation. However, a new paradigm is set to quantify the rehabilitation phase using IMU (inertial measurement unit) for the TUG test [45]. The research is still in an incipient phase, but it can provide crucial factors that helps in distinguishing the recovery rates. In the case of upper limb neuro-rehabilitation, the researchers pay more attention to automatic assessment systems as they are described in the systematic review [46]. They proposed a framework of automated assessment rehabilitation systems that will be more autonomous and objective.

Table 8 clearly emphasizes the idea that the TUG test and 6 or 10 min walking test are mostly considered for evaluation. However, a new paradigm is set to quantify the rehabilitation phase using IMU (inertial measurement unit) for TUG test [55]. The research is still in an incipient phase, but it can provide crucial factors that helps in distinguishing the recovery rates. In the case of upper limb neuro-rehabilitation, the researchers pay more attention to automatic assessment systems as it is described in the systematic review [56]. They proposed a framework of automated assessment rehabilitation systems that will be more autonomous and objective.

## 4. Conclusions

In the context of fast development of wearable technologies, more and more solutions for diagnosis, rehabilitation, assessment, and monitoring of patients with Parkinson’s disease have been discussed and presented in the scientific literature. This systematic review is comprised of two parts. The first one regards a quantitative analysis in which we collected more than 3000 articles from four databases, based on the PRISMA technique. We used a PEDro inspired method to reduce our set to 46 worthy articles. Furthermore, in the second part, a qualitative analysis has been realized based on recent solutions developed for PD diagnosis and rehabilitation. Considering all the factors and research works, we can conclude that motor disability of Parkinson’s patients significantly reduces the QoL as it is not diagnosed at initial stages and the clinical diagnoses are based on UPDRS scaling and other clinical systems scoring that proved to be limited by individual assessment and patients’ status. The existing wearable technology is playing a leading role in terms of treatment, diagnosis, and motor activity improvement of PD.

Researchers have proposed many different wearable solutions for monitoring and diagnosis of PD by putting more efforts in finding the most dominant features during gait activity such as heel off, step length, stride length, stride time, and plantar pressure. The most suitable wearable sensor devices for finding these features are wearable insoles, IMU based monitoring systems attached at lower limb, smart bands, EMG based devices, Actigraphs, GAITrite, ActivPals, and gait monitoring system using a smart-phone equipped with inertial sensors. Among all these, the insoles proved to be more dominant and useful, suggesting that these wearable solutions must be exposed to a larger population for validity. From the selected articles, it is demonstrated that repetitive intense motion activities proved to be effective for PD patients especially to those with severe motor disabilities. Great advantages are observed with treadmill and sensory motor training’s but still the results depend on the optimal location, amount of training under supervision, mode of delivery, intensity of exercise, and the type of training required to get the benefits.

With respect to rehabilitation evaluation, the TUG, 6MWKT, and 10MWKT tests are traditional gold standard examination tests for monitoring gait deflation, providing treatment analysis such as of physical therapy and various exercises. However, these tests are time consuming and task performance related, and the results are affected by many multiple variables like walking area, task complexity assigned to patient, physical exertion by participant, and incapability to finish the task as a result of fatigue. Among these, the TUG test has been used for decades, but currently new methods are being introduced from which the automatic assessment system brings remarkable results. These wearable devices help not only for rehabilitation exercises and motor improvement but also in analyzing the evolution or involution of this disease. However, there is a lot of research and development work left in this healthcare area especially on the accuracy, precision, reliability, and objective support from patients and healthcare systems for validation and adoption of these wearable solutions.

As a high number of initiatives in the previous research shows that most of the wearable devices use uni-modal sensor or bi-modal sensors, in the future, we will investigate the effectiveness of the multi-modal sensor approach for the assessment of PD. Our next directions will also focus on the role of invasive sensors. The aforementioned technology in which sensors reside inside the patient body has been proved to be very useful providing continuous information for monitoring and also diminishing PD effects like tremor or bradykinesia. Hence, we will investigate the articles regarding the impact of invasive sensors on PD patients, challenges in operation of minimally invasive sampling techniques, their application to larger community for telemedicine or telehealthcare, and their useful deployment in healthcare industry.

## Figures and Tables

**Table 1:** Numerical database search results.

Combination of Keywords	Elsevier	IEEE Xplorer	PubMed	Other Sources	Total
wearable sensors AND parkinson’s disorder	864	90	145	30	1129
wearable sensors AND neurocognitive disorder	116	0	22	12	150
parkinson patients AND rehabilitation exercises	1431	32	458	60	1981
Total	2411	122	625	102	3260

**Table 2:** Step 2: Relevant articles after screening titles and abstracts.

Elsevier	IEEE Xplorer	PubMed	Other Sources	Total
281	97	342	20	740

**Table 3:** Step 3: Relevant articles after screening of ISI Web of Science Indexing.

Elsevier	IEEE Xplorer	PubMed	Other Sources	Total
230	64	335	10	639

**Table 4:** Scoring of Q3: Number of patients/subjects participated in the study.

No. of Subjects	Q3 Ranged (0–5)	Q4 Ranged (0–1)
0	1	0.2
1	2	0.4
2–4	3	0.6
5–10	4	0.8
>10	5	1

**Table 5:** Scoring of Q4 in case of review articles.

No. of References	Q4 Ranged (0–5)	Q4 Ranged (0–1)
0	0	0
1–10	1	0.2
11–20	2	0.4
21–30	3	0.6
31–40	4	0.8
>41	5	1

**Table 6:** Motor disabilities that affects Quality of Life (QoL).

Motor Disabilities	Description
Shuffling gait	very small fleeting steps and bent postures
Freezing of gait	episodic absence in which feet are glued shut
Masked face (hypomimia)	results from unification of Bradykinesia and rigidity
Balance	inability to maintain a steady and upright posture to prevent fall
Tremor	twitching movements
Bradykinesia	slow movement
Dyskinesia	spontaneous, abnormal movements of the facial, arms, legs, or trunk
Festination	shortened and speedy steps taken during normal walking.
Rigidity	inflexibility or stiffness of joints

**Table 7:** Wearable insoles models for diagnosis, monitoring, and rehabilitation.

First Author [Ref] Year	Technology Description	Clinical Scoring System	Data Transmission Methods	Subjects	Algorithms	Features	Clinical Feature Activity	Main Results
Rosevall, J [35] 2014	Pressure and Inertial Sensors (three-axis accelerometer and three-axis gyroscope). Comfortable, flexible, portable, and suitable for clinical and home setting	Fall efficacy scale(FES), modern falls efficacy scale (MFES) and UPDRS scaling	1/2 wavelength dipole antenna and Bluetooth low energy	14	Pattern Recognition algorithm	Stride time, step length, foot clearance, and postural sway	The system analyses several gait parameters and finds patterns, markers and thresholds that differentiate between fallers and non-fallers.	They measured the fall risk. Sensors are connected between a voltage supply level and a multiplexer that can be controlled to connect one sensor at a time to the input of a transimpedance amplifier which is read using an ADC on a microcontroller that can scan the pressure distribution up to 50 times per second. The standard deviation is of order 10%.
Hatton, Anna L. [36] 2016	Smooth insoles and textured insoles worn for 14 weeks. Commercially available, inexpensive, non-invasive, and previously used in many research strategies.	Multiple sclerosis walking scale (MSWS-29), MS QoL-54 and modified fatigue impact scale	NA	176	General linear models (repeated measures analysis of variance ANOVA)	Stride length, stride time variability, double-limb support time, velocity, gait kinematics (hip, knee, and ankle joint angles, toe clearance, trunk inclination, arm swing, mediolateral pelvis), foot sensation (light touch-pressure, vibration, two-point discrimination) and proprioception (ankle joint position sense)	The results of the study suggest that the textured effect is clinically significant, the study has the potential to identify a new, evidence-based footwear intervention which has the capacity to enhance mobility and independent living in people with multiple sclerosis	This study may generate vital evidence to inform the development of more effective, multi-faceted, and multi-disciplinary rehabilitation programs, for specific gait impairments
Han, Yingzhou [37] 2016	Piezoelectric staves are inserted between the upper and lower plates on which there are wavy ribs and grooves. The force on upper plate is capable of recognizing different human movements	UPDRS, MDS-UPDRS and Unified Dyskinesia Rating Scale (UDysRS)	NA	3	Own customized algorithm	Features extracted from various kinds of voltage waveforms, which reflect variations in plantar pressure.	Forefoot and heel strike features helps in distinguishing normal and abnormal gait parameters	Monitor DLAs and the total accuracy is 93.33%, Self-detecting accuracy is 100%, and the non-self-detecting accuracy is 91.67%.
Qiu, Feng [38] 2013	Textured insoles provide a passive intervention that is an inexpensive and accessible means to enhance the somatosensory input from the plantar surface of the feet	UPDRS, MDS-UPDRS and Unified Dyskinesia Rating Scale (UDysRS)	NA	20 healthy and 20 patients	Mixed model analysis of variance (ANOVA)	Anterior posterior and medial lateral sway also standard deviation	Effect of surface standing on the foam compared to the firm surfaces (F(1,78) = 208.885, *p* < 0.001) also effect of insoles (F(2,156) = 5.825, *p* = 0.004) and post-hoc comparisons with barefoot	ML postural sway SD was greater for the PD participants compared with the control (F(1,78) = 13.165, *p* = 0.001). ML postural sway SD was not much different between the smooth and textured insoles (Fisher’s LSD: *p* = 0.127)
Mustufa, Ys Ashad [39] 2015	Multi-layered rugged, low cost, scalable and durable packaged insoles. Developed with Piezoelectric, temperature, accelerometer and force sensors	Timed up and go test (TUG)	Bluetooth communication protocol (LMX9834)	NA	NA	Plantar pressure, temperature, rotational angels of feet	The second phase will oversee the collection of a dataset for *n* = 10 healthy individuals which will be used to inform the generation of a key feature set.	The system records the plantar pressure, temperature, acceleration, and the rotation angle of the foot to provide an unobtrusive and ubiquitous hardware.

**Table 8:** Rehabilitation exercises and assessment tests.

First Author [Ref] Year	Exercise Type	Subjects/Patients	Duration	Evaluation Test	Conclusions
Capecci, Mariana [44] 2019	Robot assisted gait and Treadmill training (TT)	Total 96 subjects (48 with robot assisted and 48 with treadmill training)	20 sessions of 45 min gait training assisted by an end effector robot device (G-EO system or TT)	6 min walking test, TUG test, FOG questionnaire, UPDRS QoL questionnaire-39 administered before To and T1.	Results are better with robot assisted than TT
Flynn, Allyson [45] 2018	Home based exercise	PD subjects	4 sessions over 2 weeks	Pooled based analysis as outcome of exercise also correlation of score with follow ups of post intervention	Recovery in balance and gait speed with mild to moderate PD
Gordt, Katharina 2018 [46]	Wireless sensor training: 1. static; 2. dynamic; 3. Proactive balance training	8 randomized control trials (RCTs) were included	1 day (1 session) to 8 weeks (15 sessions in total)	Conventional balance training controls specific gait parameters and proactive balance measures.	Better results with steady state balance
Raccagni C. [47] 2019	Physiotherapy	Group of 10 individuals of Parkinsonian variant of multiple system atrophy and 10 subjects with PD Hoehn and Yahr stage(<=3)	5 day physiotherapy program followed by a 5-week unsupervised home based training.	Questionnaires along with gait motion analysis	Results are better with robot assisted than treadmill training
Hu, Bin [48] 2019	Wearable technology	300 patients	17 months	Detection of episodic gait freezing using Ambuloson during walking or stepping	UPDRS scoring decreased by 0.3 points
Koop, Mandy Miller [49] 2019	Aerobics	59 patients with idiopathic PD	8 week high intensive aerobic exercise	TUG test	Progress in lower limb movements
Carpinella [50] 2017	Wearable sensor based system named Gamepad operated as real time visual and acoustic feedback compared with physiotherapy	42 PD subjects randomized into experimental and control group	20 sessions training for balance and gait.	Assessed by blinded examiner with a one-month follow up. In addition, considered Berg balance scale (BBS), 10 MWT and questionnaires	Gait improvements and enhanced transfer of training effects
Taghizadeh [51] 2018	Sensory motor training (SMT) on hand and upper extremity sensory and motor function	40 patients with PD for SMT	10 sessions of SMT i.e., 5 days/week for 2 weeks	Pre- and post-testing sessions considering tactile acuity, proprioception, touch threshold, weight and texture discrimination, and haptic performance.	SMT subjects with severity levels 1 to 3 of the Hoehn and Yahr scale showed progress in sensory and motor actions
Mohammadi- Abdar [52] 2015	Smart bike	47 riders	Two algorithms that are static (inertial load) mode, or dynamic (speed reference) mode to collect data i.e., rider heart rate, cadence, and power at a high sampling rate.	Clinical tests	Effective tool in estimating the procure of new control paradigms for reforming the motor disabilities

## Abbreviations

PD	Parkinson’s disease
QoL	Quality of life
UPDRS	Unified Parkinson’s disease rating scale
FOG	Freezing of gait
DLA	Daily life activities
IMU	Inertial measurement unit
TUG	Timed up and go
